# Leak K^+^ channel mRNAs in dorsal root ganglia: Relation to inflammation and spontaneous pain behaviour

**DOI:** 10.1016/j.mcn.2012.01.002

**Published:** 2012-03

**Authors:** Barnaby Marsh, Cristian Acosta, Laiche Djouhri, Sally N. Lawson

**Affiliations:** School of Physiology and Pharmacology, University of Bristol, BS8 1TD, UK

**Keywords:** K2P mRNA, KCNKx.x, Potassium channel, DRG, TRESK, TASK

## Abstract

Two pore domain potassium (K2P) channels (KCNKx.x) cause K + leak currents and are major contributors to resting membrane potential. Their roles in dorsal root ganglion (DRG) neurons normally, and in pathological pain models, are poorly understood. Therefore, we examined mRNA levels for 10 K2P channels in L4 and L5 rat DRGs normally, and 1 day and 4 days after unilateral cutaneous inflammation, induced by intradermal complete Freund's adjuvant (CFA) injections. Spontaneous foot lifting (SFL) duration (spontaneous pain behaviour) was measured in 1 day and 4 day rats < 1 h before DRG harvest. mRNA levels for KCNK channels and Kv1.4 relative to GAPDH (n = 4–6 rats/group) were determined with real-time RT-PCR. This study is the first to demonstrate expression of THIK1, THIK2 and TWIK2 mRNA in DRGs. Abundance in normal DRGs was, in descending order:

Kv1.4 > TRESK(KCNK18) > TRAAK(KCNK4) > TREK2(KCNK10) = TWIK2(KCNK6) > TREK1 (KCNK2) = THIK2(KCNK12) > TASK1(KCNK3) > TASK2(KCNK5) > THIK1(KCNK13) = TASK3(KCNK9).

During inflammation, the main differences from normal in DRG mRNA levels were bilateral, suggesting systemic regulation, although some channels showed evidence of ipsilateral modulation. By 1 day, bilateral K2P mRNA levels had decreased (THIK1) or increased (TASK1, THIK2) but by 4 days they were consistently decreased (TASK2, TASK3) or tended to decrease (excluding TRAAK). The decreased TASK2 mRNA was mirrored by decreased protein (TASK2-immunoreactivity) at 4 days. Ipsilateral mRNA levels at 4 days compared with 1 day were lower (TRESK, TASK1, TASK3, TASK2 and THIK2) or higher (THIK1). Ipsilateral SFL duration during inflammation was positively correlated with ipsilateral TASK1 and TASK3 mRNAs, and contralateral TASK1, TRESK and TASK2 mRNAs. Thus changes in K2P mRNA levels occurred during inflammation and for 4 K2P channels were associated with spontaneous pain behaviour (SFL). K2P channels and their altered expression are therefore associated with inflammation-induced pain.

## Introduction

Pain of peripheral origin, both acute and chronic/pathological, results from increased CNS input from primary afferent neurons. Peripheral inflammation, and neuroinflammation in partially damaged nerves, causes increased spontaneous firing (SF), spontaneous foot lifting (SFL) and/or lowered nociceptive sensory and electrical thresholds in undamaged fibres (Djouhri et al., 2006; Shim et al., 2005; Wu et al., 2001, 2002).

The existence of K^+^ leak currents (IK) was proposed by Goldman (1943) and Hodgkin and Huxley (1952). The channels underlying these are the two pore domain potassium (K2P) leak channels. They are mainly responsible for setting resting membrane potential (Em) and are constitutively open at rest, generally voltage independent, and responsive to many factors (Enyedi and Czirjak, 2010; Lotshaw, 2007; Plant et al., 2005). Thus, through setting Em, K2P channels strongly influence neuronal excitability and firing (Brickley et al., 2007; Meuth et al., 2003). Their expressions in sensory/nociceptive neurons may therefore prove important both normally and in chronic/pathological pain states (Es-Salah-Lamoureux et al., 2010).

The K2P channels are encoded by the K2P (originally KCNK) family of genes; 15 distinct isoforms have been cloned, with only 12 apparently functional (Bayliss and Barrett, 2008). K2Ps are grouped and named in families according to functional properties: TWIK, weak inwards rectifiers; THIK, halothane inhibited; TREK, lipid, stretch and temperature activated; TASK, acid inhibited; TALK, alkaline activated; and TRESK, Ca^2+^ activated (Bayliss and Barrett, 2008; Bittner et al., 2010; Goldstein et al., 2005). Some (e.g. TRESK) are inhibited by arachidonic acid while others (e.g. TRAAK, TREK2) are both activated by it and also by GPCR (G-protein coupled receptor) agonists (Mathie, 2007). Thus, far from being passive, K2P channels are acutely modulated by ligands or environmental factors, resulting in altered leak K^+^ current, and thus altered Em.

Previous mRNA studies found high levels of TRESK, and variable levels of TRAAK, TREK1, TREK2, TWIK1 and TWIK2 in rodent (rat or mouse) DRGs (Dobler et al., 2007; Talley et al., 2001). However, each study included only few K2P channels, so all comparisons of abundance are incomplete. In addition, there has been no published indication of the extent of inter-animal variability of K2P mRNA expression.

Resting IK (and thus Em) in cultured neonatal rat DRG neurons is largely dependent on TREK2, TRESK, TRAAK, and TREK1 (Kang and Kim, 2006). It is important therefore to determine their expression in adult DRGs in vivo since expression may change in culture and also during development.

There is growing evidence for K2P channels being implicated in nociception and pain. TREK1 is co-localised with TRPV1 in some nociceptive DRG neurons and its mRNA expression is reduced after inflammation in neurons innervating the colon (La and Gebhart, 2011). Also, TREK1 knockout reduces inflammation-induced mechanical and thermal hyperalgesia (Alloui et al., 2006). TRESK knockout enhances DRG neuron excitability (Tulleuda et al., 2011), and a dominant negative TRESK mutation is implicated in migraine (Lafreniere et al., 2010). However, neither K2P mRNA expression levels in DRGs supplying inflamed tissues, nor their relationship to inflammation-induced spontaneous pain behaviour have been studied.

Accordingly, we used real-time RT-PCR to measure relative mRNA abundances and variability in expression between 4 and 6 rats/group, for 10 K2Ps in normal adult DRGs. We examined whether expression of these channels changes following inflammation induced by intradermal complete Freund's adjuvant (CFA), whether such changes are ipsilateral or bilateral, and whether they are related to spontaneous pain behaviour.

## Results

### Relative abundance in normal DRGs

**Fig. 1:**
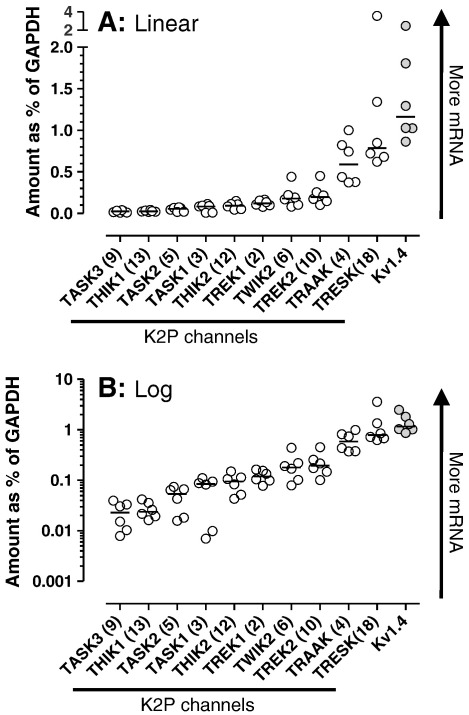
Relative abundance of K2P mRNAs in normal DRGs. All values relative to GAPDH (see Experimental methods section). Note relative amount increases upwards on the Y axis. See Experimental methods section for details of calculation. Data are from L4 and L5 DRGs from each of 6 rats, thus 6 data points. Medians are shown. A: plotted with linear Y scale, B: plotted on logarithmic Y scale.

The relative abundances of mRNAs for the different K2P channels expressed as a percentage of GAPDH mRNA, are shown in Fig. 1 , on a linear scale (Fig. 1A) and, in order to clarify differences between medians of channels with lower abundances, also on a log scale (Fig. 1B). In declining order of median values, the relative abundance was:

Kv1.4 > TRESK(KCNK18) > TRAAK(KCNK4) > TREK2(KCNK10) = TWIK2(KCNK6) > TREK1 (KCNK2) = THIK2(KCNK12) > TASK1(KCNK3) > TASK2(KCNK5) > THIK1(KCNK13) = TASK3(KCNK9).

GAPDH mRNA could be amplified to detectable levels using a modest number of cycles (median 18.7–19, see above) while the K2P mRNA required between 6 and 16 more cycles than GAPDH, indicating much higher initial number of copies of GAPDH mRNA in the DRG than for any of the other mRNAs that we studied. Of the K2P channels we examined, the maximum mRNA expression (i.e. for TRESK) was equivalent to ~ 0.8% of the total GAPDH mRNA, and the lowest expression (TASK3) was equivalent to about 0.023% of GAPDH, a 34-fold difference in median levels between TRESK and TASK3. Reported TASK3 mRNA levels vary in different tissues but, as here, can be extremely low e.g. (Hayoz et al., 2009). Kv1.4 was more highly expressed than any of the examined K2P channels at 1.16% of GAPDH values; Kv1.4 was ~ 1.5 times as abundant as TRESK.

### Variability between rats

The individual rat values were closely grouped (Fig. 1) for most channels. In contrast, TASK1 showed four well grouped values and two somewhat lower; but there was no difference in amplification efficiency between these 6 rats (1.84 ± 0.0229, Table 2). It is therefore likely that the data reflect a real difference in TASK1 mRNA abundance between rats. The ranges of normal values varied more for some channels (TRESK, TASK1, TASK2) than others (THIK1, TREK1).

### Changes in expression with inflammation

**Fig. 2:**
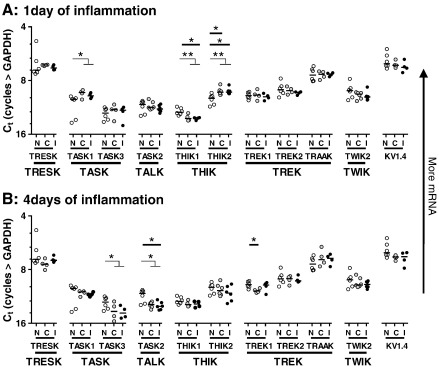
Inflammation effects on ipsilateral and contralateral K2P mRNAs. Y axis: comparative Ct analysis (Cycles > GAPDH): number of cycles necessary for K2P detection, relative to the number of cycles required for GAPDH detection in the same DRG pool; scale inverted to show mRNA increases upward. DRG data 1 day (A) and 4 days (B) after inflammation induced by CFA were compared with normal (untreated) DRG data as follows. Scatter plots with medians (1 data point per rat) were made for each channel of the following DRG data: (N), normal (bilateral), and for rats with inflammation (C) contralateral and (I) ipsilateral DRG values. Normal values are those shown in Fig. 1 and are replicated in A and B for comparison. Data ordered by K2P families (below graphs). A: 1 day of inflammation and B: 4 days of inflammation. In no case did medians differ between ipsilateral and contralateral DRGs. Statistics: Kruskal–Wallis tests (significances shown with heavy horizontal lines) with Dunn's test between N and both I and C; Wilcoxon matched pairs signed rank test between I and C (not significant); Mann Whitney test between normal values and combined ipsilateral plus contralateral (fine horizontal lines). Only significant differences shown. Significances:  *=P < 0.05, **= P < 0.01, ***= P < 0.001, ****= P < 0.0001.

**Fig. 3:**
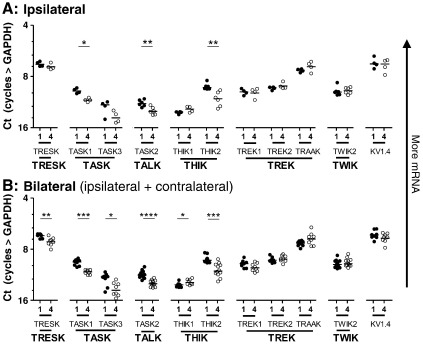
K2P mRNAs compared at 1 day and 4 days of inflammation. Y axis as described for Fig. 2. Values for mRNA for DRGs from individual rats with medians DRGs 1 day and 4 days after inflammation induced by CFA injection. Data arranged by K2P families (below graphs). In A: ipsilateral to inflammation; in B: bilateral (ipsilateral and contralateral data combined). Results for Mann Whitney tests (fine lines) between 1 day and 4 day values; significances shown as in Fig. 2.

For the following comparisons, changes in mRNA levels refer to Ct values (cycles > GAPDH) plotted with increases in mRNA (i.e. lower Ct values) being shown upwards on Y axes in Figs. 2 and 3 .

#### Ipsi- versus contralateral DRGs during inflammation (Fig. 2)

No significant differences in median mRNA levels occurred between ipsilateral and contralateral DRG (Wilcoxon matched pairs signed rank tests), for any channel examined, either 1 day or 4 days post induction of cutaneous inflammation with CFA. There were, however, some non-significant tendencies for ipsilateral mRNA values to be lower than contralateral (e.g. TRESK and TWIK2 at 1 day) or higher (e.g. TRESK and TREK1 at 4 days).

In contrast, compared to normal significant changes after inflammation were apparent ipsilaterally, contralaterally and/or bilaterally. The combination of ipsilateral and contralateral values as bilateral data was justified by the above lack of significant differences between them after inflammation; the extent to which changes occurred bilaterally may indicate systemic/global control mechanism/s. The significant changes after inflammation included the following:

*1 day of inflammation versus normal:* THIK2 was greater ipsi-, contra- and bilaterally; THIK1 was greater ipsi- and bilaterally; and TASK1 was greater bilaterally (Fig. 2A, Table 3 ).
*4 days of inflammation versus normal:* TASK2 decreased ipsi-, and bilaterally; TREK1 decreased contralaterally; TASK3 decreased bilaterally. Relative to normal, ipsilateral mRNAs were lower for most K2P channels examined (except for TRAAK) (see Fig. 2B, Table 3).
*4 days versus 1 day of inflammation:* Fig. 3 shows comparisons between values 1 day and 4 days after inflammation for ipsilateral DRGs only (Fig. 3A), and for combined ipsilateral plus contralateral (bilateral) data (Fig. 3B). 4 day mRNA levels, compared with 1 day, were lower for TASK1, TASK2 and THIK2 for ipsilateral data, and for these plus TRESK and TASK3 for bilateral data. TRESK was also lower contralaterally, see Table 3. 4 day values were higher than 1 day for only 1 channel, THIK1, significant for bilateral data only. Overall significant differences between 4 days and 1 day were seen for 6 K2P mRNAs including the most abundant (TRESK) and the 5 least abundant in this study (Fig. 1) TASK3, THIK1, TASK2, TASK1 and THIK2. Thus the trend was for these K2P mRNAs to be lower after 4 days than after 1 day of inflammation, with only THIK1 being higher at 4 days. In the TASK family, mRNA levels increased by 1 day and decreased by 4 days relative to normal, resulting in greater differences between 4 days and 1 day than between either 4 days or 1 day and normal.

Significance and direction of all the changes shown in Figs. 2 and 3, plus any contralateral changes, are summarised in Table 3.

#### Fold/percentage changes in ipsilateral mRNA during inflammation

**Fig. 4:**
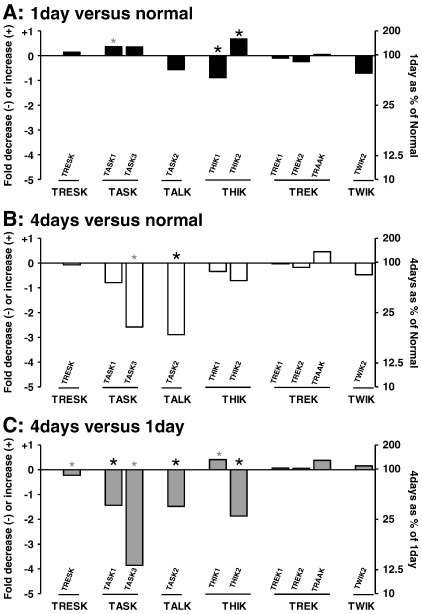
K2P mRNA: fold/percentage changes with inflammation in ipsilateral DRGs. For each channel, changes are between medians for that channel. These are expressed relative to normal values for 1 day of inflammation (A) and 4 days of inflammation (B) or 4 days relative to 1 day values (C). Because A and B are both relative to normals and C is relative to 1 day, values of A + B approximate but do not exactly match values in C. Left Y axis shows fold increases (+) and fold decreases (−) with 0 indicating no change. For comparison, the right Y axis shows these changes as a percentage with no change being 100% of normal (A, B) or of 1 day values (C). For example: − 2 (left Y axis) is a twofold decrease equivalent to 25% (right Y axis) and + 1 (left) means a 1 fold increase or 200% (right Y axis) of the initial amount of mRNA. Black asterisks show changes that are significant ipsilaterally, and grey smaller asterisks show changes that are significant only for ipsilateral plus contralateral data.

The changes described above are displayed in Fig. 4 as fold, or percentage, changes relative to normal mRNA levels for each channel.

1 day after inflammation, ipsilateral THIK1 and THIK2 exhibited the largest significant fold changes relative to normal with a 0.9 fold (~ 50%) decrease for THIK1 and ~ 0.7 fold (70%) increase in THIK2 (Fig. 4A). Non-significant changes from normal at 1 day (Fig. 4A) included increased ipsilateral TRESK (0.15 fold), TASK1 and TASK3 (both ~ 0.4 fold) and lower TASK2 and TWIK2 (0.6 and 0.7 fold decreases respectively) (Fig. 4, Table 3).

After 4 days of inflammation, most channel mRNAs tended to decrease compared with normal apart from TRAAK (see above and Fig. 4B). The greatest fold changes were in TASK3 (2.6 fold decrease) and TASK2 (nearly 3 fold decrease). These are both K2P channels with relatively low abundance normally (see Fig. 1).

At 4 days compared to 1 day of inflammation, there was lower mRNA in 5 channels (Fig. 4C) with nearly 4 fold less TASK3 and 1–2 fold reductions in TASK1, TASK2 and THIK2. TRESK mRNA showed little change (0.22 fold decrease) ipsilaterally.

#### Protein expression

**Fig. 5:**
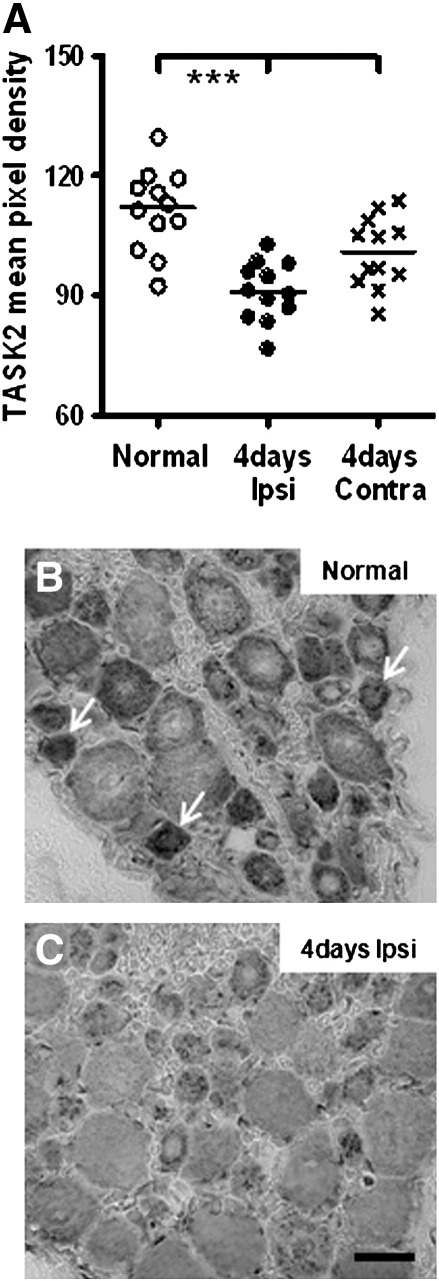
TASK2 protein expression after 4 days of inflammation versus normal. A, Comparison of the pixel density for TASK2 staining in mid-sections of L4 DRGs from untreated (normal) rats and ipsi- and contralateral L4 DRGs from rats 4 days after CFA-induced inflammation. The mean pixel densities for 3 neuron-enriched areas per animal (n = 12 per condition) are plotted. Medians were highly significantly decreased by 4 days ipsilaterally relative to normal only (*** = P < 0.001). B, Representative X40 bright field images of TASK2 DRG immunostaining normally and ipsilateral to the inflammation 4 days after CFA-injection (C). Arrows indicate stronger immunostaining in small DRG neurons and also the Golgi-type pattern of staining seen with this antibody. Scale bar represents 40 μm.

We tested whether the changes in mRNA level translate into a change in the corresponding channel protein expression by examining the immunohistochemical staining intensity for the channel with the greatest percentage change compared with normal that we saw in this study. That is TASK2 which exhibited the largest fold change at 4 days ipsilaterally relative to normal mRNA (see Fig. 4). Fig. 5 shows that the median pixel density for TASK2 in total DRG tissue was significantly lower ipsilaterally at 4 days than in the normal rats, as expected given the large decrease in mRNA in total DRG tissue at this time. Furthermore, pixel densities for the contralateral 4 day DRGs were intermediate between normal and ipsilateral 4 day DRGs, which was also the case for the mRNA; for neither the mRNA not the protein did the ipsilateral and contralateral 4 day levels differ significantly.

TASK2 staining appears to be mostly neuronal. Within neurons it was mainly cytoplasmic, has a Golgi-type pattern of staining and appears stronger in small DRG neurons (arrows in Fig. 5B). There was also indication of nuclear staining in some neurons. By 4 days after inflammation was induced by CFA injection, TASK2 immunostaining had decreased in all neuronal sizes (Fig. 5C).

### Spontaneous foot lifting (SFL) versus K2P mRNA levels

We next examined whether SFL was related to mRNA expression for any of the K2P channels we studied. The reasons were as follows a) Em is largely controlled by leak K^+^ currents associated with K2P channels; b) both neuronal excitability and firing are dependent on resting Em; c) SFL is correlated with the rate of spontaneous firing (SF) in C-fibre nociceptors, and both SFL and C-nociceptor SF are greater at 1 day than 4 days of inflammation (Djouhri et al., 2006).

**Fig. 6:**
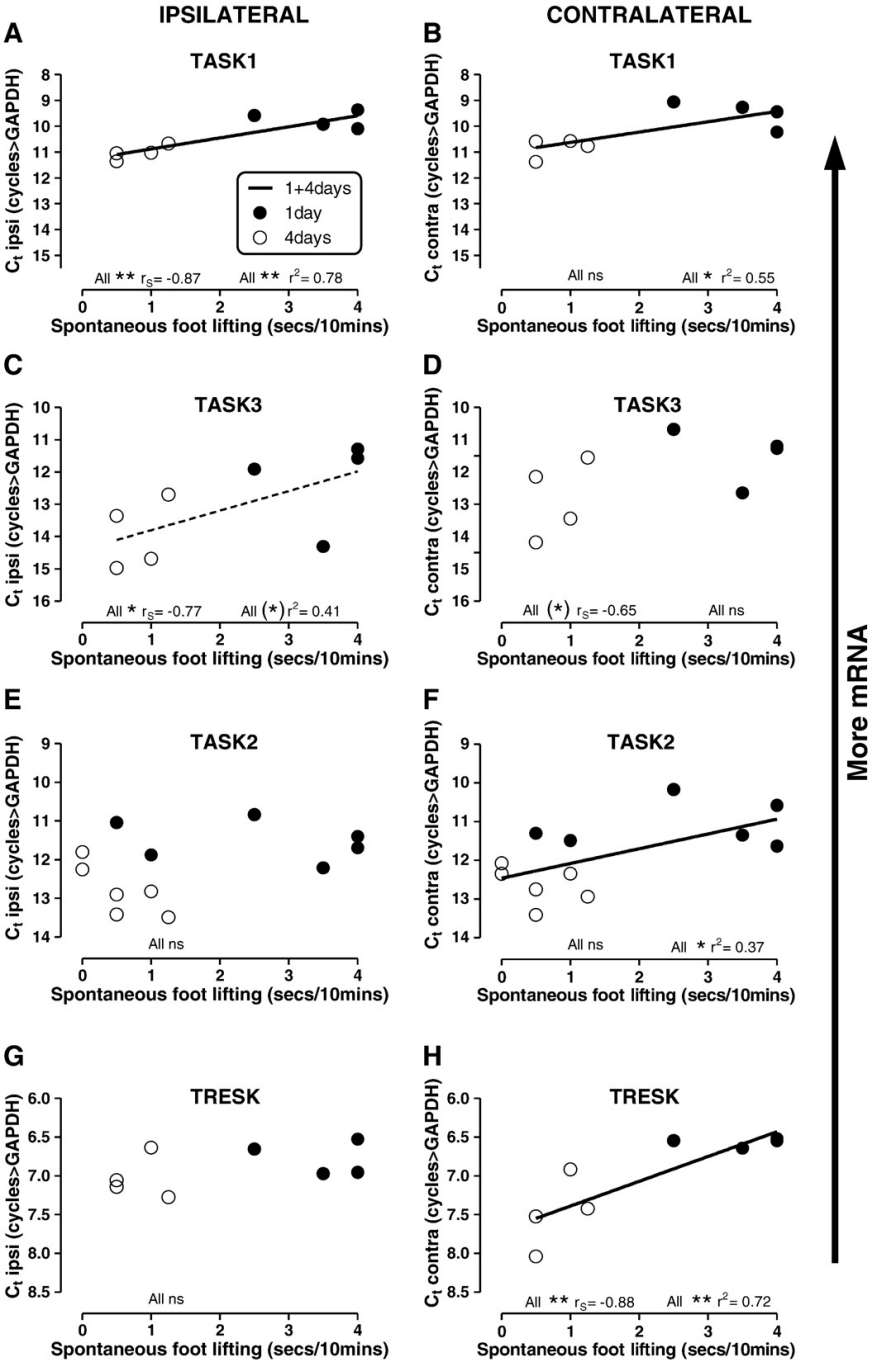
K2P mRNA correlations with spontaneous foot lifting (SFL) during inflammation. SFL duration recorded just prior to DRG removal plotted against mRNA levels 1 day (filled circles) and 4 day after inflammation induced by CFA injection (open circles). SFL was greater 1 day than 4 days after inflammation induction (P < 0.05). Note that because of their lower SFL, 4 day rats (open circles) are to the left. All correlations of SFL duration were positive with mRNA levels (negative with Ct values). SFL was correlated with TASK1 mRNA, both, ipsilaterally and contralaterally (A and B). Ipsilateral TASK3 mRNA was correlated with SFL; also dashed linear regression line indicates 0.09 > P > 0.05 (C). Contralateral TASK3 shows a similar (not significant) trend (D). TASK2 and TRESK mRNA levels were unrelated to SFL ipsilaterally (EGG) but were positively correlated with SFL contralaterally (F and H). Statistics: beneath each graph, at left are non-parametric P (significance levels) and r_est_ (Spearman's correlation coefficient); at right are linear regression values, P and r^2^. Significance levels: (* ) with dashed regression line = 0.05 > P < 0.1;  * = P < 0.05;   ** = P < 0.001.

SFL was limited to the ipsilateral hindpaw, and its duration was significantly higher at 1 day than 4 days of inflammation (P < 0.05), as previously shown (Djouhri et al., 2006). For all channels, we examined whether there were correlations between SFL duration and a) ipsilateral or b) contralateral mRNA levels (inverse of Ct values). We report only correlations that were significant for data from 1 day plus data from 4 days. Significant correlations (non-parametric Spearman's test and/or linear regression analysis) were seen between SFL and levels of four of the K2P channel mRNA levels; all were positive. Thus in all cases more mRNA was associated with more SFL. For TASK1, correlations were with ipsi- and contralateral mRNA levels but were stronger with ipsilateral levels. For TASK3 the only significant correlation was with ipsilateral levels. Only contralateral TASK2 and TRESK mRNA levels were correlated with SFL, with the correlation for TRESK being the stronger (Fig. 6 ).

## Discussion

This is the first study of relative expression of mRNA for members of all 6 K2P families in DRGs both normally and following peripheral inflammation. It is also the first report of inter-animal variability for these mRNA levels. We provide evidence for modulation of expression of 6 K2P mRNAs during inflammation, and for correlations between mRNA levels for 4 K2P channels and spontaneous pain behaviour. Our data therefore suggest an involvement of K2P mRNA expression in inflammation and/or inflammation-induced pain.

### DRG neurons versus non-neuronal cells

Purified DRG neuron cultures are useful for examining neuronal K2P mRNA expression in vitro (Kang et al., 2005; La et al., 2006; Tulleuda et al., 2011). In contrast, we studied normal expression and inflammatory-related changes in intact whole DRGs, to preserve the in vivo environment, including appropriate levels of e.g. trophic factor, inflammatory mediators etc., something that is hard to replicate in culture. DRGs contain non-neuronal cells which can express K2P channels (Pasler et al., 2007) although K2P mRNA from DRGs is probably predominantly neuronal because: a) in rodents, neurons plus fibres are a large proportion of DRG volume; b) K2P mRNA is generated in neuronal somata to provide channels for very extensive fibre plus soma membrane; c) for several K2Ps (including TASK3, TRAAK), in situ hybridisation DRG images suggest normally higher mRNA in neurons than other cells (Talley et al., 2001), also TASK2-immunocytochemistry is largely neuronal (this study) and d) relative expression of K2P mRNAs in DRGs (this study) accords with DRG neuron in situ *hybridisation* signal intensities (see below).

### Relative amounts of K2P channels in normal DRGs

Overall, the present mRNA levels relative to GAPDH are consistent with previous reports (Enyedi and Czirjak, 2010). They are also consistent with in situ hybridisation signals for these channels in normal rodent DRG neurons (Talley et al., 2001, 2003), which were strong for TRAAK and TASK2, intermediate for TASK1, TREK2, and TREK1 and present, but in very few neurons for TASK3. Because of different PCR comparators used and small numbers of channels per study we cannot compare the present full range of K2P mRNA abundances with published PCR studies. Nonetheless, our data are consistent with mRNA abundances that are high for TRAAK and low for TASK3 in rodent DRGs (Talley et al., 2001) and human DRGs (Medhurst et al., 2001) and also with “a relatively strong signal” for TRESK mRNA in rodent DRGs (Kang and Kim 2005). Our finding that TRESK and TREK2 mRNAs are amongst the most abundant in rat DRGs fit with these being major background K^+^ channels in rat DRG neurons (Dobler et al., 2007; Kang and Kim, 2006). We report for the first time mRNA expression for THIK1, THIK2 and TWIK2 in rat DRGs. Although the functions of TWIK2 are unclear, its relatively high abundance (4th highest) raises questions about possible functions in DRGs.

### K2P channel mRNA and inflammation

Our findings of altered mRNA expression of several (≥ 6) K2P channels in DRGs after inflammation are novel, apart from a previous report of TREK1 mRNA downregulation in DRGs after colon inflammation (La and Gebhart, 2011). Despite a growing understanding of K2P protein intracellular trafficking (Mathie et al., 2010), little is known about mechanisms that control/modulate K2P mRNA expression.

Surprisingly most of the significant changes in mRNA expression occurred bilaterally (at 1 day TASK1 and THIK2 increased and THIK1 decreased, while at 4 days TASK3 and TASK2 decreased) suggesting more global influences. Further experiments are needed to determine whether these are bilateral or global/systemic, as well as the nature of these influences, and whether they affect mainly neurons or other cells. Systemic influences have been shown to result from circulating inflammatory mediators (e.g. cytokines) and/or hormones released in response to inflammation or pain (Koltzenburg et al., 1999; Milligan et al., 2003; Uceyler et al., 2009). Thus at least some of these changes seem likely to result from systemic influences.

We also found evidence of differences between ipsilateral and contralateral DRG mRNA levels, although none was significant, possibly due to small rat numbers. Examples are greater changes contralaterally than ipsilaterally (increase 1 day; decrease 4 days) in TRESK mRNA, and significantly decreased TREK1 contralaterally but not ipsilaterally at 4 days. If contralateral changes result from systematic/bilateral influences, ipsilateral DRGs should be subject to these same influences. Thus differences between ipsilateral and contralateral levels suggest ipsilateral influences on mRNA expression. Because nerve fibres provide the only continuous link between ipsilateral DRGs and ipsilateral inflamed tissues, firing or transport of chemical factors along these fibres could provide solely ipsilateral influences. Nociceptive nerve fibres are the most likely to respond to inflammatory mediators/changes (Holzer and Holzer-Petsche, 2009; Lawson, 2005; Linley et al., 2010; Oliveira Fusaro et al., 2010; Schaible et al., 2011). Neurally mediated effects may involve only subpopulation/s of DRG neurons that a) respond to inflammatory mediators and b) express that particular K2P mRNA. Thus, at 4 days any differences between ipsilateral and contralateral levels for TREK1 and TRESK could result from upregulation in an ipsilateral neuronal subpopulation.

Lower expression of 5 K2P mRNAs, and higher expression of THIK1 at 4 days compared to 1 day post-CFA injections, could perhaps relate to lower levels of inflammatory mediators at 4 days (see later).

Overall, we find that expression levels of ≥ 6 mRNAs for K2P channels, on which Em depends, show complex changes in vivo during inflammation. The mechanisms influencing their expression are so far unknown.

The greatest reduction in mRNA levels seen in this study (TASK2 at 4 days) was supported by decreased TASK2-immunointensity suggesting decreased TASK2 protein levels in total DRG tissues at this time. This loss of TASK2 protein is a further confirmation of a) the downregulation of TASK2 during inflammation and b) the changes in mRNA levels serving as predictors of protein changes.

### K2P mRNA expression and inflammatory pain

The following increase in inflamed tissues: NGF, extracellular acidification (Kress et al., 1997; Reeh and Steen, 1996), inflammatory mediators like arachidonic acid (AA) (Hu et al., 2003; Samad et al., 2002) and pro-inflammatory cytokines (Koller et al., 1997; Miller et al., 2009) including TNFα (Woolf et al., 1997). Many of these acutely activate specific receptors on nociceptors causing firing e.g. see review (Lawson, 2005). CFA-induced cutaneous inflammation (this model) causes afferent C-fibre SF, SFL, allodynia and hyperalgesia that are all greater at 1 day and reduce by 4 days after injections, similar to tissue levels of pro-inflammatory mediators (Djouhri et al., 2006; Linley et al., 2010; Okun et al., 2011; Woolf and Costigan, 1999; Xie et al., 2006). SFL is correlated with C-nociceptor spontaneous firing (SF) rate and is a measure of C-fibre generated spontaneous pain (Djouhri et al., 2006). The likelihood of neuronal firing could be altered by K2P channels that affect Em, and are also acutely modulated by inflammation-related changes. We therefore examined whether K2P mRNA levels and SFL duration were related.

Our finding that mRNA levels, either ipsilateral (TASK3) or contralateral (TASK2, TRESK) or both (TASK1), were correlated with ipsilateral SFL duration suggests that mRNA expression for these channels is influenced by the extent of inflammation (since SFL is related to this) either a) globally/systemically and/or b) ipsilaterally (via sensory neurones), see above. It is also consistent with levels of K2P mRNA influencing SF and SFL, because greater K2P mRNA would result in higher K^+^ leak currents, if followed by translation to protein, appropriate trafficking, insertion into the membrane and channel activation. Note that direct responses of nociceptive fibres to local tissue inflammation, and any contribution of altered K2P expression to these responses, would be ipsilateral, consistent with ipsilateral SFL only. Our data cannot indicate whether higher K2P channel expression would stabilise the Em, or enhance neuronal activation due to local inflammation; this may differ with neuronal type and K2P channel type. However, they do suggest that determining how expression of different K2P mRNAs is controlled may be essential for a full understanding of the mechanisms underlying inflammatory pain.

### K2P channels that show modulation

Some patterns emerge in the properties of the four channels with mRNA levels that are correlated with SFL during inflammation. For example, mRNAs for TASK1, TASK3 and TASK2 were reduced at 4 days of inflammation, and all are acutely inhibited (closed) in response to acid pH (Baumann et al., 2004). All three have been implicated, and show altered expression, in inflammation associated with autoimmunity and a range of pathological conditions, involving hypoxia and decreased pH (Bittner et al., 2010). Furthermore, these 3 channels are functionally associated and often co-expressed (Gonzalez et al., 2009; Meuth et al., 2003). This is interesting because inflammation-associated acid pH would result in closure of these channels which could cause depolarisation, enhancing the likelihood of firing ipsilaterally, especially during acute (1 day) local inflammation. Whether these channels are expressed in nociceptors remains to be determined. If so, ipsilateral down-regulation of these channels by 4 days of inflammation could reduce ipsilateral nociceptor responsiveness to acid pH and contribute to the decreased C-nociceptive SF rate and SFL. The fourth channel, TRESK, is AA-inhibited, thus elevated inflammation-associated AA could depolarise TRESK-expressing nerve fibres, increasing excitability. However, firing would elevate intracellular Ca^2 + ^, activating TRESK, thus stabilising Em and limiting firing. In addition, TRESK currents are in neurons whose size distribution is similar to that of nociceptors (Kang and Kim, 2006). Thus, the properties of TRESK, its high abundance, and known importance in Em control (see earlier), suggest a potentially important (possibly protective) role for TRESK in nociceptor responses to inflammation. Interestingly a lack-of-function mutation in this channel is associated with a type of migraine (see Introduction section), suggesting this channel can indeed be protective against pain.

The physiological role of THIK1 and THIK2 “remains enigmatic” (Enyedi and Czirjak, 2010). It is therefore interesting that both were regulated during inflammation: THIK1 decreased and the more abundant THIK2 increased by 1 day; both decreased by 4 days. It is too early to speculate on possible functional significance of these changes although THIK1 current was activated by high levels of AA (Goldstein et al., 2005; Rajan et al., 2001).

The lack of significant changes or correlations with SFL for some K2P mRNAs does not exclude either post-transcriptional modulation by inflammation, or contribution to modulation of neuronal excitability or SFL by inflammation. In particular, TREK1 and TREK2, known to contribute to DRG neuron Em (see Introduction), and TRAAK, highly abundant in DRGs, may be important to include in further post-transcriptional studies.

Our findings raise many questions regarding the nature, extent and time courses of modulatory influences on K2P mRNA expression during inflammation. They also raise questions about the effects of this modulation on expression of channel protein and neuronal (especially nociceptor) membrane properties, including alterations in firing and contribution/s to inflammation-induced pain. Which K2P channels are expressed, and in which subpopulation/s of DRG neurons, also emerge as essential questions. Addressing these questions should clarify the role/s of K2P channels in sensation, nociception and inflammatory pain.

## Experimental methods

All experimental procedures complied with Home Office (United Kingdom) Guidelines. A total of 18 young female Wistar rats (140–180 g) were used throughout. Animals were culled at the end of experiments with an overdose of anaesthetic.

### Induction of cutaneous inflammation

Cutaneous inflammation in one hindlimb was induced, under isoflurane anaesthesia, by two 100 μl intradermal injections of Complete Freund's Adjuvant (CFA) (Sigma, St. Louis, MO): one into the mid-plantar surface of the left hindpaw and another lateral to the left knee as previously described (Djouhri and Lawson, 1999; Djouhri et al., 2006). DRG tissue was removed from one group of four rats after 24 h (1 day) and from a further four rats 4 days after CFA injection (4 days). Thus, in the initial study there were four 1 day and four 4 days rats and tissue from these was used for all channels studied. A further two 1 day and two 4 days rats were prepared later, and this tissue was used only for certain K2P channels. The same design was used for normal rats: the initial experiments included a group of 4 rats followed by a second group of 2 rats (total n = 6). Thus data for each channel is from the same 4 (or in some cases 6) rats. Intradermal CFA injection is a well established method of establishing inflammatory pain-related behaviour, with increased activity in nociceptive sensory neurons (Djouhri and Lawson, 1999; Djouhri et al., 2001, 2006; Millan, 1999). Although contralateral L4/5 DRGs were chosen as intra-animal controls, growing evidence suggests that circulating/systemic inflammatory influences may reach/affect all DRGs (e.g. see Jancalek et al., 2011; Koltzenburg et al., 1999). This implies that for molecules whose expression is inflammation-sensitive, intra-animal control DRGs that are completely unaffected by systemic inflammatory influences, although highly desirable, may not exist.

Comparisons of effects of CFA-induced inflammation are made with normal (untreated) rats throughout, for the following reasons. The injection will itself cause some inflammation contributing to the inflammation-induced changes that we are studying. Additionally the vehicle for CFA is mineral oil, which also generates a strong immune response (Kuroda et al., 2004). Thus comparison with vehicle-injected controls would result in comparison of a strong inflammatory influence with a milder one (vehicle injection alone), and is likely to require much larger numbers of animals to reach significance. This would be unjustified, given that we are examining the effects of inflammation, rather than the effects of CFA per se.

The choice of times (1 and 4 days) after CFA injection is based on previous studies in rat showing that spontaneous firing in nociceptors was greater at 1 day, and lower at 4 days and that spontaneous foot lifting (SFL) was also greater at 1 day than 4 days (Djouhri et al., 2006).

### Spontaneous foot lifting (SFL)

SFL was observed 1 day or 4 days after CFA injection. Observations were completed within 1 h prior to DRG harvest. SFL was measured after exploratory and grooming behaviour and locomotion had ceased and the rat was stationary on all four paws (for more detail see (Yoon et al., 1996; Djouhri et al., 2006)). SFL was the cumulative duration of time (in seconds) for which the rat elevated the ipsilateral hindpaw, measured over two 5 minute intervals. This was often accompanied by foot shaking/licking or other aversive behaviour. The contralateral (untreated) hindpaw showed no SFL. Normal (untreated) rats do not show SFL (Djouhri et al., 2006). The duration of SFL has been shown to be positively correlated with rate of C-fibre firing, rather than being secondary to allodynia (Djouhri et al., 2006).

### Choice of channels

In this study we examined 10 K2P channels including at least one channel from each of the 6 K2P families. We excluded two of the three K2P channels that have failed to give raise to functionally active channels when expressed heterologously either in *Xenopus* oocytes or mammalian cell lines (mainly COS-7 and HEK293). Those are KCNK7/8 — a member of the TWIK-family (Bockenhauer et al., 2000; Salinas et al., 1999) and KCNK15 (part of the TASK-family, now called TASK5) (Ashmole et al., 2001; Kim and Gnatenco, 2001). The third non-functional K2P channel, KCNK12 (also known as THIK2) (Rajan et al., 2001) was included because it has been linked to deafness and possibly other sensory deficiencies (Aller and Wisden, 2008; Cui et al., 2007). We excluded TWIK1 for which there is conflicting evidence regarding its functionality (Goldstein et al., 1998; Lesage et al., 1996; Pountney et al., 1999), but included the closely related TWIK2 channel, whose rat variant has been well characterised in heterologous systems (Patel et al., 2000). For the TALK family, we examined TASK2 and not TALK1 and TALK2 because a) unlike TALK1 and TALK2, TASK2 has been shown to be functionally active in several tissues of the nervous system and b) TASK2 is physiologically similar to both TALK1 and TALK2 (Enyedi and Czirjak, 2010).

### RNA extraction

Rats were killed with an overdose of 60–80 mg/kg of pentobarbital, and DRGs were removed and frozen on dry ice within 5 min. For normal (untreated) rats, L4 and L5 DRGs were extracted from both sides and processed separately. For 1 day and 4 day rats, DRG removal occurred within ~ 1 h of completing SFL observations. After the mRNA extraction was completed, the mRNA for L4 and L5 DRGs from each rat were pooled, with a separate pool for each rat. Both L4 and L5 DRGs project to the sites of CFA injections, that is, the plantar surface of the hind foot, and lateral to the knee (unpublished observations in this laboratory by Watkins). The frozen DRGs were placed in an eppendorf tube with 500 μl Trizol (Invitrogen), homogenised with an eppendorf pestle, and then triturated with multiple passages through a 25G needle and left to stand for 10 min at room temperature. The homogenate was then added to a tube containing heavy phase lock gel (Eppendorf) then 100 μl of chloroform was introduced and the mixture was agitated for ~ 5 s. It was then centrifuged at 13,000 rpm for 10 min at room temperature. The upper phase was removed with a needle and placed in a fresh tube, and the remainder was discarded. An amount of 70% ethanol, equivalent to the volume of upper phase that had been removed, was added and the mixture inverted several times.

This solution was pipetted into an RNeasy column (Qiagen) which was spun at 10,000 rpm for 15 s. After several washes in the supplied RNeasy wash buffer (Qiagen) the RNA was eluted twice with 30 μl of RNAse free water. For normal rats, L4 and L5 mRNA extracts from both sides were pooled resulting in 4 DRGs for one rat in a volume of 120 μl. For each CFA treated rat, the mRNA was retained in two pools, contralateral (L4 + L5) and ipsilateral (L4 + L5). Thus each 2 DRG pool was in a final volume of 60 μl. Thus all pools had the same ratio of DRGs to volume of extraction (4/120 μl for normals, 2/60 μl for CFAs) and therefore similar RNA concentrations.

The integrity and mRNA concentrations of the samples were assessed using a spectrophotometer (Nanodrop, USA). To this end, the 28S:18S rRNA ratio was obtained, calculated from the fluorescence readouts at 260 and 280 nm. rRNA ratio for our samples fluctuated between 1.7 and 2 showing that they had good to high integrity and did not contain RNA lysing impurities (Schoor et al., 2003; Wang et al., 2000). Concentrations of RNA were typically between 100 and 200 ng/μl. The samples were then frozen on dry ice and stored at − 80 °C until required.

### cDNA generation

Qiagen Quantitect Reverse Transcriptase Kit was employed to generate the cDNA in this study. RNA from each sample was used in three separate reactions; two Reverse Transcription (RT +) reactions and one control (RT −) reaction, which was identical except for the omission of RT and its replacement with an equal volume (1 μl) of water. To eliminate genomic DNA contamination in each reaction, 2 μl of template RNA was mixed with 2 μl of gDNA wipeout buffer and 10 μl of H_2_O in a PCR tube. This was incubated in a PCR machine at 42 °C for 2 min and then placed immediately at 4 °C. To this a pre-mixed solution containing 4 μl of Quantiscript RT buffer and 1 μl of RT primer mix (consisting of oligo-dT and random primers in water) was added, followed by 1 μl of Reverse Transcriptase (or H_2_O for the RT-reactions) for a total reaction volume of 20 μl. The resultant solution was then incubated in a PCR machine for 15 min at 42 °C, 3 min at 95 °C and frozen at − 20 °C until needed.

### Real-time RT-PCR

We conducted our experiments in accordance with recently published guidelines for quantitative PCR (qPCR) (Bustin et al., 2010). The mRNA levels of normal rats were assessed relative to glyceraldehyde-3-phosphate dehydrogenase (GAPDH) mRNA; its frequent use as a baseline reference has been well documented (see below). K2P channels investigated were TASK1 (KCNK3), TASK2 (KCNK5), TASK3 (KCNK9), TREK2 (KCNK10), THIK1 (KCNK13), THIK2 (KCNK12), TWIK2 (KCNK1), TRESK (KCNK18) and TRAAK (KCNK4); in addition, and for comparison, Kv1.4 was also examined. A detailed list of the primers used including sequences and amplicons is provided in Table 1 . Primers were reconstituted to a final concentration of 500 ng/ml and frozen at − 20 °C until required. The solutions were mixed according to the Qiagen protocols, such that each well of an ABI 96 well optical reaction plate (Applied Biosystems, USA) contained 10 μl of Quantifast Sybr Green, 7 μl of H_2_O, 2 μl of the primer pair solution and 1 μl of cDNA for a total reaction volume of 20 μl. The completed plate was sealed with an optical adhesive cover (Applied Biosystems, USA) and loaded into an ABI PRISM 7900 Sequence Detection System (Applied Biosystems, USA) programmed with standard cycling conditions (95 °C for 10 min followed by 50 cycles of 95 °C for 15 s and 60 °C for 60 s). This was followed by dissociation curve analysis to confirm the formation of specific products.

The C_t_ values (the number of cycles required for a baseline fluorescence to be detectable) were calculated by the supplied software (SDS 2.0), and the relative levels of the target gene against the endogenous GAPDH control were calculated using the comparative Ct (− ΔΔC_t_) method (Giulietti et al., 2001). GAPDH is a common housekeeping gene with high endogenous levels that has previously been used for K2P channels in rodent DRGs using the ΔΔC_t_ method (Lin et al., 2004). GAPDH remains unchanged in DRGs following inflammation of the tissues to which a DRG projects e.g. (Kerr et al., 2007; Pezet et al., 2006; Xiao et al., 2002). Furthermore, GAPDH has specifically been shown to remain unchanged in L4/5 DRGs 1, 4, 7 and 14 days after inflammation induced by CFA injection into hindfoot plantar skin (Abbadie et al., 2003; da Costa et al., 2010; Molliver et al., 2005). This is important because the primary assumption in the ΔΔC_t_ method is that the amount of reference gene does not significantly change with treatment. In this study mRNA levels are all relative to GAPDH which has been shown to be unchanged during inflammation. Because we have followed the published guidelines for minimising sources of variability in qPCR methods (see Bustin et al., 2010), these should not affect comparative Ct data. Nonetheless, our data should be interpreted with the same caution that should be applied to all qPCR data.

### Analysis of PCR data

#### Amplification efficiency

Efficiency (E) was estimated from the fluorescence increases in the exponential phase of the amplification plot (log scale) (Peirson et al., 2003; Pfaffl, 2006; Ramakers et al., 2003). In contrast to other indirect methods such as the sigmoidal and logistic curve fits, this approach is independent of background fluorescence. It is especially suitable for applications using SYBR Green I (such as our study) because this fluorophore exhibits low and constant background fluorescence. We report the overall calculated slope plus the mean E ± SE (standard error) for K2P channels normally and for the 2 channels showing the largest changes 1 and 4 days after inflammation in Table 2 . The primer efficiency values obtained were highly preserved for each channel with very similar slopes in 6 independent replications per KCNK channel irrespective of their relative abundance. Values for all individual normal rats fell within 0.1 units of the mean E for the channel, suggesting very similar efficiencies for all primers (Table 2).

Regarding differences between runs, GAPDH abundance exhibited very low Ct variability and consistent high E values (1.86 to 1.89 in all rats in all conditions). This suggests that the housekeeping gene amplification was highly preserved and did not introduce any consistent bias in the direction of changes exhibited by KCNK genes. Even for channels with the highest differences between treatments (THIK2 and TASK2), efficiency for K2P mRNAs did not differ between controls, 1 day and 4 days after inflammation (Table 2). Together the above show that differences between treatments were not due to difference in efficiency but reflect differences in mRNA abundance.

#### Amount relative to GAPDH

The raw RT RT-PCR cycle data from each DRG were treated as log_2_ of amount of transcript relative to GAPDH in that DRG. This is because each reaction cycle duplicates the number of copies from the previous cycle. These values were made negative (so that higher numbers represent more mRNA). They were then unlogged, multiplied by 100 and plotted as scatter plots on linear (Fig. 1A) and log_10_ (Fig. 1B) scales. These graphs provide an indication of the relative amount of transcript for each channel as a percentage of the amount of GAPDH in the same DRG.

#### Changes in DRG mRNA after cutaneous inflammation induced by CFA

The amounts of mRNA relative to GAPDH are expressed in subsequent figures using comparative C_t_ (cycle threshold) analysis: cycles necessary for detection, relative to the number of cycles required for GAPDH detection in the same DRG sample (C_t_ (cycles > GAPDH)). So a C_t_ of 12 for a given K2P means that 12 cycles more than needed to detect GAPDH mRNA were required for significant detection of that K2P mRNA. The Y axes are reversed in order that, as for Fig. 1, greater amounts of mRNA (fewer cycles > GAPDH) are shown upwards on the Y axis of Figs. 2, 3 and 5. In Fig. 5, these values were plotted against SFL. The number of cycles required for detection of GAPDH mRNA was compared for the TRAAK PCR as an example. Median values for all RT + samples were 19 for normal and 18.7 for both 1 day and 4 days after CFA-induced inflammation, with no consistent or significant difference between these groups or between ipsilateral and contralateral samples.

The fold increases/decreases reported in Fig. 4 were derived directly from the difference in the median C_t_ for ipsilateral 1 day rats, ipsilateral 4 day rats and untreated normal rats for each channel.

### Immunohistochemistry

#### Tissue preparation

Four untreated and four 4 day CFA-induced inflammation rats were deeply anaesthetised with pentobarbitone (80 mg/kg) and transcardially perfused with 0.9% saline at RT followed by Zamboni's fixative (Stefanini et al., 1967). L4 DRGs were then dissected and post-fixed for 20 min in Zamboni's at RT and then kept in 30% sucrose overnight. The following day serial 7 μm cryostat DRG (transverse) sections were cut. This tissue was kept at − 20 °C until used for immunoassay.

#### Immunostaining

Mid-sections of L4 DRGs were processed for staining against TASK2 using the avidin–biotin complex (ABC) immunocytochemistry method following previously published protocols (Fang et al., 2006). A 1:200 rabbit generated anti-TASK2 antibody (Chemicon, Temecula) was used for overnight incubation at 4 °C. This affinity purified antibody is directed against an epitope corresponding to amino acid residues 483–499 of human TASK-2 (Accession O95279) that is absent from any other K2P channel. The antibody has been characterised by Western blots (Chemicon) and preabsorptions (Rau et al., 2006). Furthermore, immunostaining is lost in HEK293 cells over-expressing TASK2 after a 24 h treatment with siRNA selective TASK2 knockdown (unpublished). 3, 3′-diaminobenzidine (DAB) was used to generate a coloured reaction product. No staining resulted in the absence of the primary antibody. All sections were developed simultaneously and for the same time duration to enable comparisons of relative staining intensity. Elite ABC kit and DAB reagents were from Vector Laboratories (Peterborough, UK).

#### Image analysis

Bright field images of whole DRG sections were captured with a Leica DMRBE microscope at × 5 magnification. Mean grey level intensity for three representative areas of similar size (~ 7.5 × 10^4^ μm^2^) were measured with Image J software in a single mid-section per rat per condition (making a total of 12 measurements per condition: untreated/normal, and 4 days contralateral and ipsilateral). The areas chosen were neuron-rich rather than fibre rich, for consistency. Images at × 40 were also captured to show examples of TASK2 staining.

### Statistical tests

Non-parametric tests were used throughout to compare medians, due to the small number of data points per group. Ipsilateral values were compared with contralateral values with the Wilcoxon matched pairs signed rank test. Comparisons of two groups were with the Mann–Whitney test. Because of the lack of significant difference between ipsilateral and contralateral K2P mRNA values following inflammation, ipsilateral and contralateral values could be combined as bilateral values. For comparison of more than two groups, Kruskal–Wallis tests were used, with Dunn's post-hoc tests. Correlations were with Spearman's non-parametric test, and/or linear regression analysis. All tests were made using Graphpad Prism 5 software.

## Figures and Tables

**Table 1:** Primer sequences for qRT-PCR of K2P channels.

Gene^a^	Common name	Detected transcript	Forward primer	Reverse primer	Amplicon sequence
KCNK2	TREK1	NM_172041	GTGGAGGACACATTTATTAAGT	GAAGAGGACACAGCCAAACA	GTGGAGGACACATTTATTAAGTGGAATGTTAGTCAGACCAAGATTCGTATCATCTCGACCATCATCTTCATCCTGTTTGGCTGTGTCCTCTTC
KCNK3	TASK1	NM_033376	TCATCACCACAATCGGCTAT	AGCGCGTAGAACATGCAGAA	TCATCACCACAATCGGCTATGGTCATGCGGCTCCCAGCACGGACGGAGGCAAGGTGTTCTGCATGTTCTACGCGCT
KCNK4	TRAAK	NM_053804	TGTAGGCTTTGGCGATTATGT	TGAGGCCACCCATCTCT	TGTAGGCTTTGGCGATTATGTACCAGGCGATGGCACCGGGCAGAACTCTCCAGCCTACCAGCCGCTGGTGTGGTTCTGGATCCTGTTTGGCCTAGCCTACTTCGCCTCAGTGTTCACCACTATCGGCAACTGGTTGCGAGCGGTGTCCCGCCGAACTCGCGCAGAGATGGGTGGCCTCA
KCNK5	TASK2	NM_001039516	CTATTCCTTCATCACCATCTC	AGCCCCAGGTAGATCCAAA	CTATTCCTTCATCACCATCTCCACCATTGGCTTTGGGGACTTTGTGGCCGGTGTGAACCCCAGTGCCAACTACCACGCCCTCTACCGCTACTTTGTAGAGCTTTGGATCTACCTGGGGCT
KCNK6	TWIK2	NM_053806	CACAGTCATTTTCTCCTCCTA	GCAAGAGAGTGAGATACAGAAG	CACAGTCATTTTCTCCTCCTATCTTTTCTCCCATTTCTTCTCAGACTCTGACCGTTTGGGCTTTCTGACTTTGTGAGAGTGGTGGTTTCTATGCCTTTTTGTTTTGTTTTGTTCTTGTTCTTCTGTATCTCACTCTCTTGC
KCNK9	TASK3	NM_053405	CCTTCTACTTCGCTATCAC	CCAGCGTCAGAGGGATAC	CCTTCTACTTCGCTATCACTGTCATCACAACTATCGGATATGGACATGCTGCACCTGGAACCGATGCTGGCAAGGCCTTCTGTATGTTCTATGCTGTGCTGGGTATCCCTCTGACGCTGG
KCNK10	TREK2	NM_023096	GCTGTCCTCAGTATGATT	CTTTGATCTCACCCACCTCTT	GCTGTCCTCAGTATGATTGGAGACTGGCTGCGAGTTTTATCCAAAAAGACAAAAGAAGAGGTGGGTGAGATCAAAG
KCNK12	THIK2	NM_022292	TCCTGTTCTTCAACCTCTTTCT	TGATACACCGAGGGCTT	TCCTGTTCTTCAACCTCTTTCTGGAGCGCATCATCTCGCTGCTGGCCTTCATCATGCGCGCCTGCCGGGAGCGTCAGCTGCGCCGCAGTGGCCTGCTGCCCGCCACCTTCCGCCGAGGCTCGGCGCTGTCGGAGGCCGACAGCCTGGCGGGCTGGAAGCCCTCGGTGTATCA
KCNK13	THIK1	NM_022293	TTTAACGTCATCTCCATCCT	GGAGTCCTCTTTGGCATTG	TTTAACGTCATCTCCATCCTGATCAAACAGACAGTGAACTGGATCCTGAGGAAACTGGATAGCGGGTGCTTCCCACAATGCCAAAGAGGACTCC
KCNK18	TRESK	NM_001003820	CTCACTTCTTCCTCTTCTTCTC	TAGCAAGGTAGCGAAACCTCT	CTCACTTCTTCCTCTTCTTCTCCATCTACATCATCGTGGGCATGGAGATCCTGTTCATCGCCTTCAAACTGATGCAGAACCGGCTCCTGCACACCTACAAAACCCTCATGCTGTTTGTTTGCCAAAGAGAGGTTTCGCTACCTTGCTA
KCNA4	Kv1.4	NM_012971	CACAGAGAGACTGAAAACGAA	CCCAGGGAAGAAGAAGTAGA	CACAGAGAGACTGAAAACGAAGAACAGACCCAGCTGACCCAAAACGCAGTCAGTTGCCCATACCTACCTTCTAATTTGCTCAAGAAATTTCGGAGCTCTACTTCTTCTTCCCTGGG
GAPDH	GAPDH	NM_017008	CGCATCTTCTTGTGCAGT	AATGAAGGGGTCGTTGATGG	CGCATCTTCTTGTGCAGTGCCAGCCTCGTCTCATAGACAAGATGGTGAAGGTCGGTGTGAACGGATTTGGCCGTATCGGACGCCTGGTTACCAGGGCTGCCTTCTCTTGTGACAAAGTGGACATTGTTGCCATCAACGACCCCTTCATT

a According to the International Union of Pharmacology.

**Table 2:** Mean amplification efficiencies (E) and slopes for different mRNAs.

Normal			1 day contra			1 day ipsi	
Channel	Overall slope	E (mean) ± SE	Channel	Overall slope	E (mean) ± SE	Overall slope	E (mean) ± SE
GAPDH	0.2751	1.88 ± 0.0102	TASK2	0.2710	1.87 ± 0.0114	0.2787	1.90 ± 0.0125
TRESK	0.2688	1.86 ± 0.0173	THIK2	0.2678	1.85 ± 0.0201	0.2683	1.86 ± 0.0277
TRAAK	0.2760	1.89 ± 0.0082					
TREK1	0.2688	1.86 ± 0.0256	4 days contra			4 days ipsi	
THIK1	0.2769	1.89 ± 0.0234	Channel	Overall slope	E (mean) ± SE	Overall slope	E (mean) ± SE
TREK2	0.2580	1.82 ± 0.0184					
TASK1	0.2624	1.84 ± 0.0229	TASK2	0.2739	1.88 ± 0.0268	0.2718	1.87 ± 0.0308
TASK3	0.2683	1.87 ± 0.0201	THIK2	0.2676	1.85 ± 0.0246	0.2750	1.88 ± 0.0049
TASK2	0.2720	1.87 ± 0.0132					

**Table 3:**
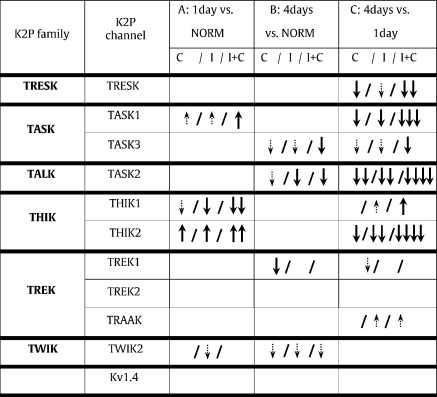
Summary of significant changes in K2P mRNA after inflammation induced by CFA: In all columns C/I/I + C refers to contralateral (C), ipsilateral (I) and ipsilateral plus contralateral (bi-lateral) DRGs (I + C). Comparisons are between Ct values shown in Figs. 2 and 3. Column A: 1 day compared to normal values, B: 4 day rats compared to normal, and C: 4 days compared with 1 day. Columns A, B: results of a) Kruskal–Wallis tests as in Fig. 2, between normals, I and C DRGs for each treatment, and b) Mann–Whitney tests results between normal and combined I plus C data, since in no case did I and C medians differ. Column C: Mann–Whitney test results (data on Fig. 3). Black thick arrows denote statistically significant differences; smaller dotted arrows indicate trends. Down arrows indicate decrease, up arrows show increase. Numbers of black arrows indicate levels of significance (↓:P ≤ 0.05; ↓↓:P ≤ 0.01; ↓↓↓:P < 0.001; ↓↓↓↓P < 0.0001). 4 day values were lower than 1 day for several channels (TRESK, TASK1, TASK2 and THIK2). Only if I and C DRGs were pooled were the following significant: 4 day < 1 day (TASK3); and 4 day > 1 day (THIK1).
